# Who do treatments work for and why? Understanding treatment-effect mechanisms in stratified medicine

**DOI:** 10.1186/1745-6215-14-S1-O10

**Published:** 2013-11-29

**Authors:** Richard Emsley, Hanhua Liu, Sabine Landau, Graham Dunn

**Affiliations:** 1 The University of Manchester, Manchester, UK; 2 Institute of Psychiatry, King's College London, London, UK

## 

The development of stratified medicine depends on an understanding of treatment-effect mechanisms (effects on therapeutic targets that mediate the effect of the treatment on clinical outcomes). Yet the evaluation of these mechanisms is often absent from the design and analysis of studies for stratified medicine, and even if present, is subject to unmeasured confounding.

We review the problem of confounding (common causes) for the drawing of valid inferences concerning treatment-effect mechanisms, even when the data has been generated using a randomised controlled trial. We illustrate the potential of the predictive biomarker-stratified trial design, together with baseline measurement of all known prognostic markers, to enable us to evaluate both the utility of the predictive biomarker in such a stratification and to estimate how much of the treatment's effect is actually explained by changes in the putative mediator. We call this a biomarker-stratified efficacy and mechanisms evaluation (BS-EME) trial design.

The analysis strategy involves the use of instrumental variable estimation methods, using the treatment by predictive biomarker interaction as an instrumental variable together with adjustments for all know prognostic markers; the latter contributing to increased precision (as in a conventional analysis of treatment effects) rather than bias reduction. The analysis approach provides unbiased estimates even in the presence of unmeasured confounding.

We conclude that stratification without corresponding mechanisms evaluation lacks credibility and in the almost certain presence of mediator-outcome confounding, mechanisms evaluation is dependent on stratification for its validity. Our trial design and analysis approach evaluates both stratification and treatment-effect mediation.

